# Supplementary material for: Weak evidence of country- and institution-related status bias in the peer review of abstracts
Source: eLife. 2021 Mar 18;10:e64561. doi: 10.7554/eLife.64561 (PMC8009675; doi:10.7554/eLife.64561)
Supplement: Supplementary file 1. [file elife-64561-supp1.docx]

***Meta-Research: Weak evidence of country- and institution-related status bias in the peer review of abstracts***

**Supplementary file 1**

**Qualtrics survey: Astronomy (page 1)**

**The six abstracts used in the survey experiment (page 10)**

**Email invitation (page 13)**

**Qualtrics survey: Astronomy**

**Consent form**

 **Purpose of this research study**: We investigate how researchers assess scientific abstracts and use them to determine what literature to read.

 **What you will do in the study**: We will ask you to read and assess one scientific abstract. The abstract has been adapted to suit the purposes of this study and will be of relevance to your research field. We will also ask you to complete some questions about yourself and your research field. 

 **Time required**: This survey typically takes about 10 minutes to complete.

 **Risks**: There are no anticipated risks in this study.  

 **Benefits**: You will not directly benefit from participating in this study. The study will contribute to research on evaluative judgment in science.

 **Privacy and confidentiality:** Should you agree to take part, your participation will be completely confidential. We will collect contact information and publicly available publication data from the bibliographic database Web of Science. Contact information (name, affiliation and e-mail) will be used to link publication data to the respondents. This data will only be collected to construct aggregate publication profiles. No analyses will target the individual level. All information gathered in the survey will be stored securely in compliance with the standards set by the Danish Data Protection Agency and are fully compliant with the European Union General Data Protection Regulation (GDPR). No one outside the research group will have access to the data. Data will be kept by the research group until the analyses are finalized, at the latest in January 2021. After this point, all data will be completely anonymised. Participation in the study is completely voluntary. This study has been approved by Aarhus University's Institutional Review Board. 

 **Right to withdraw from the study**: If at any time you do not wish to continue your participation, you are welcome to withdraw from the survey without penalty. 

 **How to withdraw from the study**: You can end your participation by closing the browser window. If you want to withdraw after completing the survey, please contact Dr. Jens Peter Andersen or Dr. Mathias Wullum Nielsen (see email information below).

 **Questions?**
 For questions, concerns, or complaints, please contact Associate Professor Jens Peter Andersen (jpa@ps.au.dk) or Associate Professor Mathias Wullum Nielsen (mwn@soc.ku.dk).

 **Participate**
 If you wish to participate in this study, please click the button that says “I agree to participate”. You will then be directed to complete the survey. Otherwise, please close the browser window.

- I agree to participate

**End of Block: Consent**

**Start of Block: AbstractHH3**

We would like you to read through the abstract below.

On the next page we will ask you six questions about your immediate impressions of the abstract. 
__________________________

 **Accurate fundamental parameters of solar-like stars**

 Douglas Little & Joel Sawyer, Harvard University, USA (1)

Asteroseismology provides a valuable window into the internal structure and dynamics of stars. Detailed modeling of asteroseismic observations from the Transitioning Exoplanet Survey Satellite (TESS) requires accurate fundamental stellar parameters. The available parameters are often quite uncertain, with deviations as extreme as 0.5 dex in [Fe/H] and 1 dex in log g between standard catalogue values and results from high-resolution spectroscopy. This study seeks to derive accurate metallicities for main sequence stars. We do so by combining spectroscopic analysis of medium resolution spectra (with moderate signal-to-noise) with constraints from multi-color photometry and measurements of average seismic quantities from the TESS light curves. Using our spectral synthesis method SPECSYNTH in conjunction with this data, we derive accurate metallicities for 1207 targets, reaching a mean uncertainty of 0.14 dex on [Fe/H]. Further, we analyze 1438 artificially degraded spectra. This analysis yields results consistent with those of our original high resolution, high signal-to-noise spectra (mean offset of -0.04+-0.08 dex on [Fe/H]). Our paper shows that basic constraints from asteroseismology and multi-color photometry can be used to derive accurate metallicities from medium resolution spectra with a moderate-to-low S/N. This approach significantly reduces the analysis overhead in spectral synthesis, and increases the follow-up efficiency of TESS targets by large, ground-based surveys like LAMOST and GALAH.

 (1) Center for Astrophysics, Harvard & Smithsonian, 60 Garden Street, Cambridge, MA 02138, USA
  __________________________
 
When you are done reading, please press the button below. **You will not be able to return to the abstract.**

Timing

First Click

Last Click

Page Submit

Click Count

**End of Block: AbstractHH3**

**Start of Block: Outcomes**

| 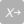 | 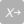 |
| --- | --- |

Researchers regularly skim through scientific abstracts to determine what papers to read. Here, we are interested in your immediate impressions of the abstract that you just read. 
 We understand that the specific topic of the abstract may not match your exact field of research. We also acknowledge that it may be difficult to evaluate the presented research based on the limited information provided in the abstract, but please give us your best estimate.
Please rate the abstract on the following four dimensions:

|  | Very Poor | Poor | Fair | Good | Excellent |
| --- | --- | --- | --- | --- | --- |
| Originality of the presented research |  |  |  |  |  |
| Credibility of the results |  |  |  |  |  |
| Significance for future research |  |  |  |  |  |
| Clarity of the abstract |  |  |  |  |  |

| 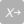 | 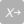 |
| --- | --- |

Imagine you were doing a research study on solar-type stars and came across this abstract online. 
**Would, or would you not consider opening the full text and continue reading?**

- I would consider opening the full text and continue reading
- I would not consider opening the full text and continue reading

Imagine the following hypothetical scenario: You are a member of the scientific committee of a large, international astronomy conference.
 The conference received a high number of abstracts of varying quality for oral presentations.
 It is necessary to limit the number of presentations to about 25% of the submissions.
 **As a reviewer, would or would you not recommend this abstract for an oral presentation in the conference programme?**

- I would recommend it for oral presentation
- I would not recommend it for oral presentation

Timing

First Click

Last Click

Page Submit

Click Count

**End of Block: Outcomes**

**Start of Block: Demographics**

| 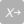 | 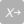 |
| --- | --- |

**Now we will ask you to answer some questions about yourself and your research field.

 Which of the following options best resemble your current position level as a researcher?**

- PhD Student (or equivalent)
- Postdoc
- Assistant professor (or equivalent)
- Associate professor (or equivalent)
- Professor (or equivalent)
- Technical or administrative staff with research time (research assistant, lab assistant, staff statistician)
- Technical or administrative staff without research time
- Other, please specify ________________________________________________

| 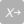 |
| --- |

**In which country do you currently reside?**

▼ Afghanistan ... Zimbabwe

| 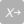 | 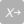 |
| --- | --- |

**In which country were you born?**

▼ Afghanistan ... Zimbabwe

| 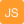 | 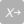 | 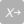 |
| --- | --- | --- |

**In which year were you born?**

▼ 2000 ... 1919

| 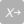 |
| --- |

**Do you have a PhD (or equivalent) degree?**

- Yes
- No

*Display This Question:*

*If Do you have a PhD (or equivalent) degree? = Yes*

| 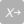 |
| --- |

**In which year did you receive your PhD (or equivalent) degree?**

▼ 2019 ... 1950 or earlier

| 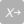 |
| --- |

**What is your gender?**

- Woman
- Man
- Non-binary
- None of the above
- Prefer not to answer

| 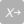 | 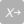 |
| --- | --- |

**Astronomy covers a broad range of research topics.
 How close would you say that the topic addressed in the abstract is to your own research area?**

- Extremely close
- Very close
- Somewhat close
- Not too close
- Not close at all

| Page Break |  |
| --- | --- |

| 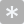 |
| --- |

**Think of this ladder as representing where researchers stand in your research field.** People define research fields in different ways, please define it in whatever way is most meaningful to you. 
At the top of the ladder are the people who have the highest standing in their research field. At the bottom are the people who have the lowest standing in their research field. 
 **Think about yourself as a researcher. Where would you place yourself on this ladder?**
 (On mobile devices you might have to click twice. You should see a green box.)


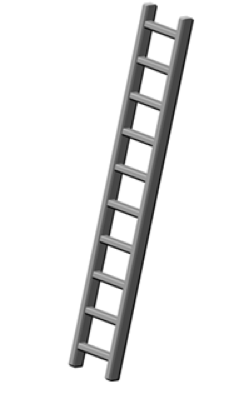


**End of Block: Demographics**

**Start of Block: Meritocracy questions**

| 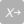 | 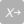 |
| --- | --- |

**Below are three statements about peer evaluation and recognition in your research field.** **Please rate your level of agreement or disagreement with each statement.**

|  | Strongly agree | Agree | Neither agree nor disagree | Disagree | Strongly disagree |
| --- | --- | --- | --- | --- | --- |
| In my research field, scientists evaluate research primarily on its merit, i.e. according to accepted standards of the field |  |  |  |  |  |
| In my research field, peer evaluation is fair and objective |  |  |  |  |  |
| In my research field, research will receive the recognition it deserves, regardless of who produced it |  |  |  |  |  |

**End of Block: Meritocracy questions**

**Start of Block: Manip1**

| 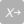 | 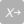 |
| --- | --- |

Finally, we are curious about what type of information researchers find most memorable when reading an abstract.
 Please answer the following question about the abstract you just read.

 **Were the authors of the abstract located at Massachusetts Institute of Technology, Columbia University or Harvard University?**

- Massachusetts Institute of Technology
- Columbia University
- Harvard University
- None of the above
- Don't know

**End of Block: Manip1**

**Start of Block: Manip2and3**

| 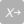 | 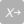 |
| --- | --- |

Finally, we are curious about what type of information researchers find most memorable when reading an abstract.
 Please answer the following question about the abstract you just read.

 **Were the authors of the abstract located at a university in the United States, Israel, Greece or Brazil?**

- The United States
- Israel
- Greece
- Brazil
- None of the above
- Don't know

**End of Block: Manip2and3**

**Thank you for participating in this study. Your participation is greatly appreciated.**

As was previously mentioned in the consent form, the purpose of the study was to investigate how researchers assess scientific abstracts. Specifically, we were interested in examining how information about authors' institutional affiliations and national affiliations may influence the way their work is perceived by scientific peers.

To investigate this, we have asked an international sample of researchers in your field to assess the exact same abstract, but with different information in the abstract’s byline. This design will allow us to determine whether the institutional and national affiliation of researchers influence the amount of attention their work receives. In this study, we hypothesize that scientific peers, on average, will rate the abstract more positively when it is introduced by researchers from highly visible research institutions and scientific nations. All analyses will be carried out at an aggregate level. Our study design does not allow us to make any inferences about how individual participants’ perceptions of the abstract may have been influenced by information about the authors' affiliation.

As was previously mentioned in the consent form, the abstract presented earlier was adapted for this study. The author information in the abstract’s byline is fictitious and has been made-up for the purposes of this study. We could not provide you with all of these details prior to your participation, as we wanted to ensure that your reactions in this study were spontaneous and not influenced by prior knowledge about the specific hypotheses tested. This study has been approved by Aarhus University's Institutional Review Board. 

Your participation in this survey is completely confidential. All information gathered in the survey will be stored securely in compliance with the standards set by the Danish Data Protection Agency and the European Union General Data Protection Regulation (GDPR). No one outside the research group will have access to the data. Data will be kept by the research group until the analyses are finalized, at the latest in January 2021.

Now that you know more about the study, you may decide that you do not want your data used in this research. If you would like your data removed from the study and permanently deleted, please contact Jens Peter Andersen or Mathias Wullum Nielsen (see emails below).

**We kindly ask you not to disclose the research procedures and hypotheses to anyone who might participate in this study in the future, as this could affect the results of the study.**

If you have any questions or concerns regarding this study, its purpose or procedures, please feel free to contact Associate Professor Jens Peter Andersen (email: jpa@ps.au.dk, phone: +4587165891) or Associate Professor Mathias Wullum Nielsen (email: mwn@soc.ku.dk).

If you would like to learn more about how institutional affiliations and country affiliations may influence scholarly judgment, please see the following references:

Ross, J. S., Gross, C. P., Desai, M. M., Hong, Y., Grant, A. O., Daniels, S. R., ... & Krumholz, H. M. (2006). Effect of blinded peer review on abstract acceptance. Jama, 295(14), 1675-1680.

Tomkins, A., Zhang, M., & Heavlin, W. D. (2017). Reviewer bias in single-versus double-blind peer review. Proceedings of the National Academy of Sciences, 114(48), 12708-12713.

**Thanks again for your participation!**

**The six abstracts used in the survey experiment**

**Astronomy**

**Accurate fundamental parameters of solar-like stars**

Douglas Little & Joel Sawyer

Asteroseismology provides a valuable window into the internal structure and dynamics of stars. Detailed modeling of asteroseismic observations from the Transitioning Exoplanet Survey Satellite (TESS) requires accurate fundamental stellar parameters. The available parameters are often quite uncertain, with deviations as extreme as 0.5 dex in [Fe/H] and 1 dex in log g between standard catalogue values and results from high-resolution spectroscopy. This study seeks to derive accurate metallicities for main sequence stars. We do so by combining spectroscopic analysis of medium resolution spectra (with moderate signal-to-noise) with constraints from multi-color photometry and measurements of average seismic quantities from the TESS light curves. Using our spectral synthesis method SPECSYNTH in conjunction with this data, we derive accurate metallicities for 1207 targets, reaching a mean uncertainty of 0.14 dex on [Fe/H]. Further, we analyze 1438 artificially degraded spectra. This analysis yields results consistent with those of our original high resolution, high signal-to-noise spectra (mean offset of -0.04+-0.08 dex on [Fe/H]). Our paper shows that basic constraints from asteroseismology and multi-color photometry can be used to derive accurate metallicities from medium resolution spectra with a moderate-to-low S/N. This approach significantly reduces the analysis overhead in spectral synthesis, and increases the follow-up efficiency of TESS targets by large, ground-based surveys like LAMOST and GALAH.

**Cardiology**

**Obesity and adverse events in patients with incident atrial fibrillation**
Douglas Little & Joel Sawyer

Obesity has been associated with increased risk of atrial fibrillation (AF), but little is known about the relation between body mass and stroke or death in AF patients. 
**Methods:**We examined 626 patients (404 men and 222 women) diagnosed with incident atrial fibrillation in the period 2008-2014. Baseline and follow-up data for all patients were obtained from medical records at five local hospitals. We used the cox proportional hazard model to estimate the hazard ratio (HR) for the end-point with normal-weight patients as the reference group.
**Results:**282 of the participants (45%) were overweight (BMI 25 to <30), and 152 (24%) were obese (BMI ≥30). During a median follow-up of 38 months, 120 deaths and 42 thromboembolic events (98% ischemic strokes) had occurred. The risk of an adverse event, including ischemic stroke, thromboembolism, or death, was higher in obese and overweight patients compared to patients of normal weight (Obese patients: HR 1.52; 95% CI 1.12-1.92; Overweight patients: HR: 1.24, 95% CI 1.04-1.45). Subgroup analyses stratified by sex indicated that obese men and normal-weight women were at high risk of adverse events.
**Conclusion:**Obesity is a risk factor for adverse events in AF patients. The association between BMI and ischemic stroke, thromboembolism, or death in AF patients may be modified by sex.

**Materials Science**

**Cellular gene therapy**

Douglas Little & Joel Sawyer

Cellular gene therapy uses genes to treat or prevent disease. The bottleneck in manufacturing such therapies is to introduce gene-editing molecules into cells. Existing delivery technologies suffer from at least one of the following drawbacks: low efficiency, low cell viability, lack of scalability, high cost, and potential to be harmful. We show that nanostraws, hollow metal-oxide nanotubes, can be used to establish a fluidic pipeline into human hematopoietic stem cells (HSCs). This allows for high-efficiency delivery of exogenous molecules such as nucleic acids with high cell viability. To optimize delivery parameters, we used fluorescent molecules and functional reporters, such as mRNA coding, for green fluorescent protein. In doing so, we reached high efficiencies while maintaining unperturbed cell viability. With these optimized parameters, we show that nanostraw-mediated delivery of cas9-mRNA can be used to perform stable genetic edits. This makes it a scalable tool for the manufacturing of genetically engineered HSCs.

**Political Science**

**Do pre-election polls influence citizens’ engagement with political information?**

Douglas Hughes & Joel Sawyer

This study investigates how pre-election polls influence voters’ search for political information during national elections. Building on recent observational studies of the effects of poll results on voting preferences, we hypothesize that exposure to pre-election polls diminishes voters’ engagement with political information. In an experimental online setup simulating a national election campaign, we expose a nationally representative sample of 745 participants to different levels of polling information. We then track the time that each participant spends searching for information about the listed candidates and parties prior to casting their vote. We find that high levels of exposure to pre-election polls, on average, reduces the time spent by voters on political information seeking. This finding is stable across levels of education and factual political knowledge. Our findings add to discussions of how pre-election polls shape voting behavior, and suggest that an extensive focus on polls during national elections may decrease political engagement.

**Psychology**

**Associations between the Big Five and life outcomes: a lifespan approach**

Douglas Little & Joel Sawyer

Extant research demonstrates associations between the Big Five personality traits and life outcomes at the personal, interpersonal and occupational level. Here, we add to this literature by examining variations in such associations over the life course. Specifically, we examine how age may moderate associations between the Big Five personality traits and outcomes such as life satisfaction, physical and mental health, divorce rates, occupational success, and socio-demographic status. In a longitudinal study of a nationally representative sample of 2,015 subjects, with multiple waves of measurement, we observe notable age-related fluctuations in the association strength between the Big Five personality traits and life outcomes at the personal, interpersonal and occupational level. Our findings suggest that a lifespan approach may allow for more precise analyzes of how personality traits relate to life outcomes with potential implications for targeted interventions.

**Public Health**

**Socio-demographic determinants of health behavior and obesity in the elderly**

Douglas Little & Joel Sawyer

Obesity has been associated with a variety of adverse health consequences, but little is known about the socio-demographic determinants of obesity in the elderly. We examined the prevalence of obesity in an older population, and analyzed associations between socio-demographic factors and obesity and health behavior. 
**Methods:**Participants were recruited at four local health centers over 12 months. 2,739 adults between 60 and 80 years of age participated in the study, 55% of them women. The median age was 68. The obesity prevalence (BMI>30) was 15% for women and 19% for men. We measured the participants’ height, weight, and waist circumference. We collected information about socio-demographic factors (age, sex, marital status, educational level), and select health behaviors through a self-administered questionnaire.
**Results:**Multiple logistic regression analysis demonstrated associations between low education level and/or sex and obesity. High education level and being female was associated with healthy eating and regular exercise. We observed no statistically significant relationship between marital status and age group and the two outcome measures.
**Conclusion:**Future health initiatives aimed at reducing obesity in the elderly may benefit from considering socio-demographic factors, including sex and education level.

**Email invitation**

Dear XXXX,

The rapid growth in scientific publishing makes it increasingly difficult for researchers to keep up-to-date. We investigate how researchers assess scientific abstracts and use them to determine what literature to read.

Given your research in the area of XXXX, we invite you to participate in a brief survey. You will be asked to assess one scientific abstract in your field of research. Your email address has been obtained from one of your published papers.

This survey takes about 5-10 minutes to complete. You can open the survey by following this link.

[XXXXXXXXX]

**If you are not a researcher in XXXX, you are not in the target population for this study, and we apologize for the inconvenience.**

If you would like to know more about the survey please contact us (e-mail: [XXXX@ps.au.dk](mailto:XXXX@ps.au.dk) and XXX@soc.ku.dk).

Thanks in advance!

Sincerely

Jens Peter Andersen, Senior Researcher, PhD

Danish Centre for Studies in Research and Research Policy,
Department of Political Science
Aarhus University
Bartholins Alle 7

DK-8000, Aarhus, Denmark

Mathias Wullum Nielsen, Associate Professor, PhD
Department of Sociology

University of Copenhagen

Øster Farimagsgade 5

1353 Copenhagen-K
