# Supplementary material for: Weak evidence of country- and institution-related status bias in the peer review of abstracts
Source: eLife. 2021 Mar 18;10:e64561. doi: 10.7554/eLife.64561 (PMC8009675; doi:10.7554/eLife.64561)
Supplement: Supplementary file 2. [file elife-64561-supp2.docx]

***Meta-Research: Weak evidence of country- and institution-related status bias in the peer review of abstracts***

**Supplementary file 2**

**Tables S1-S50**

**Table S1.** Equivalence test for abstract rating in Astronomy (High status US vs. Lower status US)

TOST results:

t-value lower bound: 3.01 p-value lower bound: 0.001

t-value upper bound: -2.98 p-value upper bound: 0.002

degrees of freedom : 385.16

Equivalence bounds (Cohen's d):

low eqbound: -0.3

high eqbound: 0.3

Equivalence bounds (raw scores):

low eqbound: -0.6758

high eqbound: 0.6758

TOST confidence interval:

lower bound 90% CI: -0.369

upper bound 90% CI: 0.375

NHST confidence interval:

lower bound 95% CI: -0.441

upper bound 95% CI: 0.447

Equivalence Test Result:

The equivalence test was significant, t(385.16) = -2.981, p = 0.00153, given equivalence bounds of -0.676 and 0.676 (on a raw scale) and an alpha of 0.05.

Null Hypothesis Test Result:

The null hypothesis test was non-significant, t(385.16) = 0.0133, p = 0.989, given an alpha of 0.05.

Based on the equivalence test and the null-hypothesis test combined, we can conclude that the observed effect is statistically not different from zero and statistically equivalent to zero.

**Table S2.** Equivalence test for abstract rating in Astronomy (Lower status US vs. Lower status Non-US)

TOST results:

Z-value lower bound: 0.449 p-value lower bound: 0.327

Z-value upper bound: -5.22 p-value upper bound: 0.00000009

Equivalence bounds:

low eqbound: -0.12

high eqbound: 0.12

TOST confidence interval:

lower bound 90% CI: -0.171

upper bound 90% CI: -0.031

NHST confidence interval:

lower bound 95% CI: -0.184

upper bound 95% CI: -0.018

Equivalence Test based on Fisher's exact z-test Result:

The equivalence test was non-significant, Z = 0.449, p = 0.327, given equivalence bounds of -0.120 and 0.120 and an alpha of 0.05.

Null-Hypothesis Fisher's exact z-test Result:

The null hypothesis test was significant, Z = -2.384, p = 0.0171, given an alpha of 0.05.

Based on the equivalence test and the null-hypothesis test combined, we can conclude that the observed effect is statistically different from zero and statistically not equivalent to zero.

**Table S3.** Equivalence test for abstract rating in Cardiology (High status US vs. Lower status US)

TOST results:

t-value lower bound: 4.47 p-value lower bound: 0.000005

t-value upper bound: -1.54 p-value upper bound: 0.062

degrees of freedom : 392.75

Equivalence bounds (Cohen's d):

low eqbound: -0.3

high eqbound: 0.3

Equivalence bounds (raw scores):

low eqbound: -0.8197

high eqbound: 0.8197

TOST confidence interval:

lower bound 90% CI: -0.049

upper bound 90% CI: 0.849

NHST confidence interval:

lower bound 95% CI: -0.136

upper bound 95% CI: 0.936

Equivalence Test Result:

The equivalence test was non-significant, t(392.75) = -1.539, p = 0.0622, given equivalence bounds of -0.820 and 0.820 (on a raw scale) and an alpha of 0.05.

Null Hypothesis Test Result:

The null hypothesis test was non-significant, t(392.75) = 1.467, p = 0.143, given an alpha of 0.05.

Based on the equivalence test and the null-hypothesis test combined, we can conclude that the observed effect is statistically not different from zero and statistically not equivalent to zero.

**Table S4.** Equivalence test for abstract rating in Cardiology (Lower status US vs. Lower status Non-US)

TOST results:

t-value lower bound: 3.02 p-value lower bound: 0.001

t-value upper bound: -3.11 p-value upper bound: 0.001

degrees of freedom : 415.95

Equivalence bounds (Cohen's d):

low eqbound: -0.3

high eqbound: 0.3

Equivalence bounds (raw scores):

low eqbound: -0.7907

high eqbound: 0.7907

TOST confidence interval:

lower bound 90% CI: -0.436

upper bound 90% CI: 0.414

NHST confidence interval:

lower bound 95% CI: -0.518

upper bound 95% CI: 0.496

Equivalence Test Result:

The equivalence test was significant, t(415.95) = 3.024, p = 0.00132, given equivalence bounds of -0.791 and 0.791 (on a raw scale) and an alpha of 0.05.

Null Hypothesis Test Result:

The null hypothesis test was non-significant, t(415.95) = -0.0427, p = 0.966, given an alpha of 0.05.

Based on the equivalence test and the null-hypothesis test combined, we can conclude that the observed effect is statistically not different from zero and statistically equivalent to zero.

**Table S5.** Equivalence test for abstract rating in Materials Science (High status US vs. Lower status US)

TOST results:

t-value lower bound: 1.96 p-value lower bound: 0.025

t-value upper bound: -3.91 p-value upper bound: 0.00006

degrees of freedom : 380.84

Equivalence bounds (Cohen's d):

low eqbound: -0.3

high eqbound: 0.3

Equivalence bounds (raw scores):

low eqbound: -0.7738

high eqbound: 0.7738

TOST confidence interval:

lower bound 90% CI: -0.691

upper bound 90% CI: 0.179

NHST confidence interval:

lower bound 95% CI: -0.774

upper bound 95% CI: 0.262

Equivalence Test Result:

The equivalence test was significant, t(380.84) = 1.964, p = 0.0251, given equivalence bounds of -0.774 and 0.774 (on a raw scale) and an alpha of 0.05.

Null Hypothesis Test Result:

The null hypothesis test was non-significant, t(380.84) = -0.971, p = 0.332, given an alpha of 0.05.

Based on the equivalence test and the null-hypothesis test combined, we can conclude that the observed effect is statistically not different from zero and statistically equivalent to zero.

**Table S6.** Equivalence test for abstract rating in Materials Science (Lower status US vs. Lower status Non-US)

TOST results:

t-value lower bound: 1.70 p-value lower bound: 0.045

t-value upper bound: -3.92 p-value upper bound: 0.00005

degrees of freedom: 347.44

Equivalence bounds (Cohen's d):

low eqbound: -0.3

high eqbound: 0.3

Equivalence bounds (raw scores):

low eqbound: -0.7288

high eqbound: 0.7288

TOST confidence interval:

lower bound 90% CI: -0.716

upper bound 90% CI: 0.14

NHST confidence interval:

lower bound 95% CI: -0.798

upper bound 95% CI: 0.222

Equivalence Test Result:

The equivalence test was significant, t(347.44) = 1.699, p = 0.0451, given equivalence bounds of -0.729 and 0.729 (on a raw scale) and an alpha of 0.05.

Null Hypothesis Test Result:

The null hypothesis test was non-significant, t(347.44) = -1.110, p = 0.268, given an alpha of 0.05.

Based on the equivalence test and the null-hypothesis test combined, we can conclude that the observed effect is statistically not different from zero and statistically equivalent to zero.

**Table S7.** Equivalence test for abstract rating in Political Science (High status US vs. Lower status US)

TOST results:

t-value lower bound: 2.85 p-value lower bound: 0.002

t-value upper bound: -4.93 p-value upper bound: 0.0000005

degrees of freedom: 667.7

Equivalence bounds (Cohen's d):

low eqbound: -0.3

high eqbound: 0.3

Equivalence bounds (raw scores):

low eqbound: -0.6811

high eqbound: 0.6811

TOST confidence interval:

lower bound 90% CI: -0.47

upper bound 90% CI: 0.106

NHST confidence interval:

lower bound 95% CI: -0.526

upper bound 95% CI: 0.162

Equivalence Test Result:

The equivalence test was significant, t(667.7) = 2.850, p = 0.00225, given equivalence bounds of -0.681 and 0.681 (on a raw scale) and an alpha of 0.05.

Null Hypothesis Test Result:

The null hypothesis test was non-significant, t(667.7) = -1.039, p = 0.299, given an alpha of 0.05.

Based on the equivalence test and the null-hypothesis test combined, we can conclude that the observed effect is statistically not different from zero and statistically equivalent to zero.

**Table S8.** Equivalence test for abstract rating in Political Science (Lower status US vs. Lower status Non-US)

TOST results:

t-value lower bound: 3.19 p-value lower bound: 0.0007

t-value upper bound: -4.51 p-value upper bound: 0.000004

degrees of freedom: 654.72

Equivalence bounds (Cohen's d):

low eqbound: -0.3

high eqbound: 0.3

Equivalence bounds (raw scores):

low eqbound: -0.667

high eqbound: 0.667

TOST confidence interval:

lower bound 90% CI: -0.4

upper bound 90% CI: 0.172

NHST confidence interval:

lower bound 95% CI: -0.454

upper bound 95% CI: 0.226

Equivalence Test Result:

The equivalence test was significant, t(654.72) = 3.190, p = 0.000745, given equivalence bounds of -0.667 and 0.667 (on a raw scale) and an alpha of 0.05.

Null Hypothesis Test Result:

The null hypothesis test was non-significant, t(654.72) = -0.658, p = 0.511, given an alpha of 0.05.

Based on the equivalence test and the null-hypothesis test combined, we can conclude that the observed effect is statistically not different from zero and statistically equivalent to zero.

**Table S9.** Equivalence test for abstract rating in Psychology (High status US vs. Lower status US)

TOST results:

t-value lower bound: 3.62 p-value lower bound: 0.0002

t-value upper bound: -2.52 p-value upper bound: 0.006

degrees of freedom: 409.61

Equivalence bounds (Cohen's d):

low eqbound: -0.3

high eqbound: 0.3

Equivalence bounds (raw scores):

low eqbound: -0.7542

high eqbound: 0.7542

TOST confidence interval:

lower bound 90% CI: -0.27

upper bound 90% CI: 0.54

NHST confidence interval:

lower bound 95% CI: -0.348

upper bound 95% CI: 0.618

Equivalence Test Result:

The equivalence test was significant, t(409.61) = -2.523, p = 0.00601, given equivalence bounds of -0.754 and 0.754 (on a raw scale) and an alpha of 0.05.

Null Hypothesis Test Result:

The null hypothesis test was non-significant, t(409.61) = 0.550, p = 0.583, given an alpha of 0.05.

Based on the equivalence test and the null-hypothesis test combined, we can conclude that the observed effect is statistically not different from zero and statistically equivalent to zero.

**Table S10.** Equivalence test for abstract rating in Psychology (Lower status US vs. Lower status Non-US)

TOST results:

t-value lower bound: 3.54 p-value lower bound: 0.0002

t-value upper bound: -2.52 p-value upper bound: 0.006

degrees of freedom: 405.4

Equivalence bounds (Cohen's d):

low eqbound: -0.3

high eqbound: 0.3

Equivalence bounds (raw scores):

low eqbound: -0.7724

high eqbound: 0.7724

TOST confidence interval:

lower bound 90% CI: -0.29

upper bound 90% CI: 0.55

NHST confidence interval:

lower bound 95% CI: -0.371

upper bound 95% CI: 0.631

Equivalence Test Result:

The equivalence test was significant, t(405.4) = -2.520, p = 0.00606, given equivalence bounds of -0.772 and 0.772 (on a raw scale) and an alpha of 0.05.

Null Hypothesis Test Result:

The null hypothesis test was non-significant, t(405.4) = 0.510, p = 0.610, given an alpha of 0.05.

Based on the equivalence test and the null-hypothesis test combined, we can conclude that the observed effect is statistically not different from zero and statistically equivalent to zero.

**Table S11.** Equivalence test for abstract rating in Public Health (High status US vs. Lower status US)

TOST results:

t-value lower bound: 3.95 p-value lower bound: 0.00004

t-value upper bound: -2.60 p-value upper bound: 0.005

degrees of freedom: 472.29

Equivalence bounds (Cohen's d):

low eqbound: -0.3

high eqbound: 0.3

Equivalence bounds (raw scores):

low eqbound: -0.8583

high eqbound: 0.8583

TOST confidence interval:

lower bound 90% CI: -0.254

upper bound 90% CI: 0.608

NHST confidence interval:

lower bound 95% CI: -0.337

upper bound 95% CI: 0.691

Equivalence Test Result:

The equivalence test was significant, t(472.29) = -2.602, p = 0.00478, given equivalence bounds of -0.858 and 0.858 (on a raw scale) and an alpha of 0.05.

Null Hypothesis Test Result:

The null hypothesis test was non-significant, t(472.29) = 0.676, p = 0.499, given an alpha of 0.05.

Based on the equivalence test and the null-hypothesis test combined, we can conclude that the observed effect is statistically not different from zero and statistically equivalent to zero.

**Table S12.** Equivalence test for abstract rating in Public Health (Lower status US vs. Lower status Non-US)

TOST results:

t-value lower bound: 3.29 p-value lower bound: 0.0005

t-value upper bound: -3.35 p-value upper bound: 0.0004

degrees of freedom: 477.86

Equivalence bounds (Cohen's d):

low eqbound: -0.3

high eqbound: 0.3

Equivalence bounds (raw scores):

low eqbound: -0.8536

high eqbound: 0.8536

TOST confidence interval:

lower bound 90% CI: -0.432

upper bound 90% CI: 0.416

NHST confidence interval:

lower bound 95% CI: -0.514

upper bound 95% CI: 0.498

Equivalence Test Result:

The equivalence test was significant, t(477.86) = 3.286, p = 0.000546, given equivalence bounds of -0.854 and 0.854 (on a raw scale) and an alpha of 0.05.

Null Hypothesis Test Result:

The null hypothesis test was non-significant, t(477.86) = -0.0311, p = 0.975, given an alpha of 0.05.

Based on the equivalence test and the null-hypothesis test combined, we can conclude that the observed effect is statistically not different from zero and statistically equivalent to zero.

**Table S13.** Logit model predicting ‘Open full-text’ in Astronomy

|  |  | **OR** | **95% CI** | |
| --- | --- | --- | --- | --- |
|  | High status, US | 0.85 | 0.51 | 1.44 |
|  | Lower status, Non-US | 0.78 | 0.46 | 1.30 |
|  | Lower status, US (Reference) |  |  |  |
|  |  |  |  |  |
|  | Number of respondents | 597 |  |  |
|  | Log Likelihood | -283.2754 |  |  |
|  | Pseudo R^2^ | 0.002 |  |  |

**Table S14.** Logit model predicting ‘Open full-text’ in Cardiology

|  |  | **OR** | **95% CI** | |
| --- | --- | --- | --- | --- |
|  | High status, US | 1.15 | 0.76 | 1.74 |
|  | Lower status, Non-US | 1.02 | 0.68 | 1.53 |
|  | Lower status, US (Reference) |  |  |  |
|  |  |  |  |  |
|  | Number of respondents | 609 |  |  |
|  | Log Likelihood | -387.40034 |  |  |
|  | Pseduo R^2^ | 0.001 |  |  |

**Table S15.** Logit model predicting ‘Open full-text’ in Materials Science

|  |  | **OR** | **95% CI** | |
| --- | --- | --- | --- | --- |
|  | High status, US | 1.37 | 0.76 | 2.50 |
|  | Lower status, Non-US | 1.34 | 0.72 | 2.50 |
|  | Lower status, US (Reference) |  |  |  |
|  |  |  |  |  |
|  | Number of respondents | 553 |  |  |
|  | Log Likelihood | -207.44494 |  |  |
|  | Pseudo R^2^ | 0.003 |  |  |

**Table S16.** Logit model predicting ‘Open full-text’ in Political Science

|  |  | **OR** | **95% CI** | |
| --- | --- | --- | --- | --- |
|  | High status, US | 1.14 | 0.71 | 1.83 |
|  | Lower status, Non-US | 0.83 | 0.53 | 1.30 |
|  | Lower status, US (Reference) |  |  |  |
|  |  |  |  |  |
|  | Number of respondents | 1018 |  |  |
|  | Log Likelihood | -380.14953 |  |  |
|  | Pseudo R^2^ | 0.003 |  |  |

**Table S17.** Logit model predicting ‘Open full-text’ in Psychology

|  |  | **OR** | **95% CI** | |
| --- | --- | --- | --- | --- |
|  | High status, US | 1.52 | 0.90 | 2.57 |
|  | Lower status, Non-US | 1.11 | 0.67 | 1.84 |
|  | Lower status, US (Reference) |  |  |  |
|  |  |  |  |  |
|  | Number of respondents | 629 |  |  |
|  | Log Likelihood | -279.10618 |  |  |
|  | Pseudo R^2^ | 0.005 |  |  |

**Table S18.** Logit model predicting ‘Open full-text’ in Public Health

|  |  | **OR** | **95% CI** | |
| --- | --- | --- | --- | --- |
|  | High status, US | 0.74 | 0.51 | 1.09 |
|  | Lower status, Non-US | 0.63 | 0.43 | 0.92 |
|  | Lower status, US (Reference) |  |  |  |
|  |  |  |  |  |
|  | Number of respondents | 734 |  |  |
|  | Log Likelihood | -469.24563 |  |  |
|  | Pseudo R^2^ | 0.006 |  |  |

**Table S19.** Logit predicting ‘Include in conference’ in Astronomy

|  |  | **OR** | **95% CI** | |
| --- | --- | --- | --- | --- |
|  | High status, US | 0.81 | 0.55 | 1.21 |
|  | Lower status, Non-US | 0.77 | 0.51 | 1.15 |
|  | Lower status, US (Reference) |  |  |  |
|  |  |  |  |  |
|  | Number of respondents | 592 |  |  |
|  | Log Likelihood | -406.16545 |  |  |
|  | Pseudo R^2^ | 0.002 |  |  |

**Table S20.** Logit model predicting ‘Include in conference’ in Cardiology

|  |  | **OR** | **95% CI** | |
| --- | --- | --- | --- | --- |
|  | High status, US | 0.87 | 0.58 | 1.31 |
|  | Lower status, Non-US | 0.97 | 0.65 | 1.45 |
|  | Lower status, US (Reference) |  |  |  |
|  |  |  |  |  |
|  | Number of respondents | 610 |  |  |
|  | Log Likelihood | -396.23562 |  |  |
|  | Pseudo R^2^ | 0.001 |  |  |

**Table S21.** Logit model predicting ‘Include in conference’ in Materials Science

|  |  | **OR** | **95% CI** | |
| --- | --- | --- | --- | --- |
|  | High status, US | 0.88 | 0.57 | 1.35 |
|  | Lower status, Non-US | 0.98 | 0.62 | 1.54 |
|  | Lower status, US (Reference) |  |  |  |
|  |  |  |  |  |
|  | Number of respondents | 550 |  |  |
|  | Log Likelihood | -344.57837 |  |  |
|  | Pseudo R^2^ | 0.001 |  |  |

**Table S22.** Logit model predicting ‘Include in conference’ in Political Science

|  |  | **OR** | **95% CI** | |
| --- | --- | --- | --- | --- |
|  | High status, US | 0.61 | 0.41 | 0.93 |
|  | Lower status, Non-US | 0.69 | 0.45 | 1.05 |
|  | Lower status, US (Reference) |  |  |  |
|  |  |  |  |  |
|  | Number of respondents | 1009 |  |  |
|  | Log Likelihood | -464.12614 |  |  |
|  | Pseudo R^2^ | 0.006 |  |  |

**Table S23.** Logit predicting ‘Include in conference’ in Psychology

|  |  | **OR** | **95% CI** | |
| --- | --- | --- | --- | --- |
|  | High status, US | 1.12 | 0.76 | 1.65 |
|  | Lower status, Non-US | 1.07 | 0.72 | 1.59 |
|  | Lower status, US (Reference) |  |  |  |
|  |  |  |  |  |
|  | Number of respondents | 629 |  |  |
|  | Log Likelihood | -420.7869 |  |  |
|  | Pseudo R^2^ | 0.000 |  |  |

**Table S24.** Logit model predicting ‘Include in conference’ in Public Health

|  |  | **OR** | **95% CI** | |
| --- | --- | --- | --- | --- |
|  | High status, US | 0.90 | 0.59 | 1.36 |
|  | Lower status, Non-US | 0.95 | 0.63 | 1.42 |
|  | Lower status, US (Reference) |  |  |  |
|  |  |  |  |  |
|  | Number of respondents | 732 |  |  |
|  | Log Likelihood | -412.59827 |  |  |
|  | Pseudo R^2^ | 0.000 |  |  |

**Table S25.** Mixed linear model Predicting ‘abstract rating’

|  |  | **Coef.** | **SE** | **99% CI** | |
| --- | --- | --- | --- | --- | --- |
| **Fixed** | Intercept | 13.99 | 0.37 | 13.04 | 14.94 |
|  | High status, US | 0.03 | 0.10 | -0.22 | 0.28 |
|  | Lower status, Non-US | -0.01 | 0.10 | -0.26 | 0.23 |
|  | Lower status, US (Reference) |  |  |  |  |
| **Random** |  |  |  |  |  |
|  | Residual | 6.50 | 0.15 | 6.14 | 6.88 |
|  | Discipline | 0.80 | 0.47 | 0.18 | 3.61 |
|  |  |  |  |  |  |
| **Model specifications** | Number of disciplines | 6 |  |  |  |
|  | Number of respondents | 4111 |  |  |  |
|  | Log Likelihood | -9641.683 |  |  |  |

**Table S26.** Mixed logit model predicting ‘Open full-text’

|  |  | **OR** | **99% CI** | |
| --- | --- | --- | --- | --- |
| **Fixed** | Intercept | 4.15 | 2.19 | 7.84 |
|  | High status, US | 1.05 | 0.81 | 1.35 |
|  | Lower status, Non-US | 0.88 | 0.68 | 1.12 |
|  | Lower status, US (Reference) |  |  |  |
| **Random** |  |  |  |  |
|  | Dicipline | 0.28 | 0.61 | 1.31 |
|  |  |  |  |  |
| **Model specifications** | Number of disciplines | 6 |  |  |
|  | Number of respondents | 4140 |  |  |
|  | Log Likelihood | -2024.4492 |  |  |

**Table S27.** Mixed logit model predicting ‘Include in conference’

|  |  | **OR** | **99% CI** | |
| --- | --- | --- | --- | --- |
| **Fixed** | Intercept | 1.350 | 0.492 | 3.702 |
|  | High status, US | 0.858 | 0.690 | 1.067 |
|  | Lower status, Non-US | 0.896 | 0.719 | 1.116 |
|  | Lower status, US (Reference) |  |  |  |
| **Random** |  |  |  |  |
|  | Discipline | 0.750 | 0.167 | 3.363 |
|  |  |  |  |  |
| **Model specifications** | Number of disciplines | 6 |  |  |
|  | Number of respondents | 4122 |  |  |
|  | Log Likelihood | -2464.2411 |  |  |

**Table S28.** Mixed linear model Predicting ‘Abstract rating’ (Manipulation check)

|  |  | **Coef.** | **SE** | **99% CI** | |
| --- | --- | --- | --- | --- | --- |
| **Fixed** | Intercept | 14.04 | 0.39 | 13.01 | 15.05 |
|  | High status, US | -0.06 | 0.12 | -0.38 | 0.26 |
|  | Lower status, Non-US | -0.07 | 0.12 | -0.38 | 0.23 |
|  | Lower status, US (Reference) |  |  |  |  |
| **Random** |  |  |  |  |  |
|  | Residual | 5.95 | 0.17 | 5.53 | 6.40 |
|  | Discipline | 0.89 | 0.52 | 0.20 | 4.05 |
|  |  |  |  |  |  |
| **Model specifications** | Number of disciplines | 6.00 |  |  |  |
|  | Number of respondents | 2474 |  |  |  |
|  | Log Likelihood | -5729.215 |  |  |  |

**Table S29.** Mixed logit model predicting ‘Include in conference’ (manipulation check)

|  |  | **OR** | **99% CI** | |
| --- | --- | --- | --- | --- |
| **Fixed** | Intercept | 1.36 | 0.49 | 3.76 |
|  | High status, US | 0.78 | 0.58 | 1.04 |
|  | Lower status, Non-US | 0.89 | 0.67 | 1.18 |
|  | Lower status, US (Reference) |  |  |  |
| **Random** | Discipline |  |  |  |
| **Model specifications** | Number of disciplines | 6 |  |  |
|  | Number of respondents | 2476 |  |  |
|  | Log Likelihood | -1431.7806 |  |  |

**Table S30.** Mixed logit model predicting ‘Open full-text’ (manipulation check)

|  |  | **OR** | **99% CI** | |
| --- | --- | --- | --- | --- |
| **Fixed** | Intercept | 4.38 | 2.14 | 8.97 |
|  | High status, US | 1.11 | 0.78 | 1.56 |
|  | Lower status, Non-US | 0.93 | 0.68 | 1.29 |
|  | Lower status, US (Reference) |  |  |  |
| **Random** | Discipline | .34 | .07 | 1.64 |
| **Model specifications** | Number of disciplines | 6 |  |  |
|  | Number of respondents | 2486 |  |  |
|  | Log Likelihood | -1159.4489 |  |  |

**Table S31.** Mixed linear model Predicting ‘Abstract rating’ (Topic match)

|  |  | **Coef.** | **SE** | **99% CI** | |
| --- | --- | --- | --- | --- | --- |
| **Fixed** | Intercept | 14.03 | 0.42 | 12.96 | 15.11 |
|  | High status, US | 0.14 | 0.14 | -0.22 | 0.51 |
|  | Lower status, Non-US | 0.11 | 0.15 | -0.27 | 0.48 |
|  | Lower status, US (Reference) |  |  |  |  |
|  |  |  |  |  |  |
| **Random** | Residual | 6.74 | 0.21 | 6.21 | 7.32 |
|  | Discipline | 0.98 | 0.58 | 0.21 | 4.49 |
|  |  |  |  |  |  |
| **Model specifications** | Number of disciplines | 6.00 |  |  |  |
|  | Number of respondents | 1979 |  |  |  |
|  | Log Likelihood | -4707.612 |  |  |  |

**Table S32.** Mixed logit model predicting ‘Include in conference’ (Topic Match)

|  |  | **OR** | **99% CI** | |
| --- | --- | --- | --- | --- |
| **Fixed** | Intercept | 1.47 | .49 | 4.41 |
|  | High status, US | .92 | .67 | 1.26 |
|  | Lower status, Non-US | .90 | .65 | 1.25 |
|  | Lower status, US (Reference) |  |  |  |
| **Random** | Discipline | .87 | .19 | 3.95 |
|  |  |  |  |  |
| **Model specifications** | Number of disciplines | 6 |  |  |
|  | Number of respondents | 1976 |  |  |
|  | Log Likelihood | -1158.4146 |  |  |

**Table S33.** Mixed logit model predicting ‘Open full-text’ (Topic match)

|  |  | **OR** | **99% CI** | |
| --- | --- | --- | --- | --- |
| **Fixed** | Intercept | 4.56 | 2.24 | 9.31 |
|  | High status, US | 1.12 | 0.78 | 1.59 |
|  | Lower status, Non-US | .82 | 0.58 | 1.16 |
|  | Lower status, US (Reference) |  |  |  |
| **Random** | Discipline | .39 | .08 | 1.89 |
| **Model specification** | Number of disciplines | 6 |  |  |
|  | Number of respondents | 1981 |  |  |
|  | Log Likelihood | -996.11388 |  |  |

**Table S34.** Mixed logit model predicting ‘Include in conference’ (Moderation analysis, Lower status (US) vs. Lower status (elsewhere))

|  |  | **OR** | **99% CI** | |
| --- | --- | --- | --- | --- |
| **Fixed** | Intercept |  |  |  |
|  | Lower status (US) (ref= Lower status, Non-US) | 2.15 | .58 | 7.99 |
|  | Meritocracy score | 1.11 | 1.02 | 1.20 |
|  | Lower status (US)*Meritocracy score | .96 | .86 | 1.07 |
|  | Ladder score | .98 | .90 | 1.07 |
|  | Lower status (US)*Ladder score | 1.00 | .88 | 1.12 |
|  | Structural position | .99 | .60 | 1.61 |
|  | Lower status (US)*Structural position | .98 | .50 | 1.92 |
|  | Research Accomplishments | 1.23 | .76 | 1.98 |
|  | Lower status (US)*Research Accomplishments | .61 | .31 | 1.19 |
| **Random** | Discipline | .858 | 1.90 | 3.88 |
| **Model specification** | Number of disciplines | 6 |  |  |
|  | Number of respondents | 2501 |  |  |
|  | Log Likelihood | -1473.7949 |  |  |

**Table S35.** Mixed logit model predicting ‘Open full-text’ (Moderation analysis, Lower status (US) vs. Lower status (elsewhere))

|  |  | **OR** | **99% CI** | |
| --- | --- | --- | --- | --- |
| **Fixed** | Lower status (US) (ref= Lower status, Non-US) | .36 | .08 | 1.52 |
|  | Meritocracy score | 1.04 | .95 | 1.13 |
|  | Lower status (US) *Meritocracy score | 1.04 | .92 | 1.18 |
|  | Ladder score | .89 | .804 | .98 |
|  | Lower status (US)*Ladder score | 1.14 | 1.0 | 1.30 |
|  | Structural position | 1.18 | .70 | 1.99 |
|  | Lower status (US)*Structural position | .84 | .40 | 1.75 |
|  | Research Accomplishments | .94 | .57 | 1.54 |
|  | Lower status (US)*Research Accomplishments | .84 | .41 | 1.70 |
| **Random** | Discipline | .28 | .058 | 1.31 |
| **Model specification** | Number of disciplines | 6 |  |  |
|  | Number of respondents | 2509 |  |  |
|  | Log Likelihood | -1253.6911 |  |  |

**Table S36.** Mixed linear model predicting ‘Abstract rating’ (Moderation analysis, High status (US) vs. Lower status (US))

|  |  | **Coef.** | **99% CI** | |
| --- | --- | --- | --- | --- |
| **Fixed** | Intercept | 12.20 | 10.80 | 13.59 |
|  | High status (US) (ref= Lower status, US) | -.57 | -2.07 | .93 |
|  | Meritocracy score | .20 | .12 | .29 |
|  | High status (US) *Meritocracy score | -.01 | -.13 | .12 |
|  | Ladder score | -.08 | -.17 | .01 |
|  | High status (US) *Ladder score | .13 | .00 | .26 |
|  | Structural position | .55 | .05 | 1.06 |
|  | High status (US) *Structural position | -.59 | -1.33 | .145 |
|  | Research Accomplishments | -.29 | -.80 | .24 |
|  | High status (US)*Research Accomplishments | -.15 | -.87 | .58 |
| **Random** | Discipline | .80 | .18 | 3.63 |
|  | Residual | 6.14 | 5.71 | 6.61 |
| **Model specification** | Number of disciplines | 6 |  |  |
|  | Number of respondents | 2529 |  |  |
|  | Log Likelihood | -5896.1912 |  |  |

**Table S37.** Mixed linear model predicting ‘Abstract rating’ (Moderation analysis, Lower status (US) vs. Lower status (Non-US))

|  |  | **Coef.** | **99% CI** | |
| --- | --- | --- | --- | --- |
| **Fixed** | Intercept | 12.02 | 10.57 | 13.48 |
|  | Lower status (US) (ref= Lower status, Non-US) | .20 | -1.26 | 1.65 |
|  | Meritocracy score | .19 | .10 | .28 |
|  | Lower status (US)*Meritocracy score | -.02 | -.11 | .14 |
|  | Ladder score | -.01 | -.11 | .08 |
|  | Lower status (US)*Ladder score | -.07 | -.20 | .06 |
|  | Structural position | -.04 | -.57 | .49 |
|  | Lower status (US)*Structural position | .59 | -.14 | 1.32 |
|  | Research Accomplishments | -.03 | -.56 | .50 |
|  | Lower status (US)*Research Accomplishments | -.23 | -.96 | .51 |
| **Random** | Discipline | .93 | .21 | 4.22 |
|  | Residual | 6.09 | 5.66 | 6.55 |
| **Model specification** | Number of disciplines | 6 |  |  |
|  | Number of respondents | 2490 |  |  |
|  | Log Likelihood | -5794.6306 |  |  |

**Table S38.** Mixed logit model predicting ‘Open full-text’ (Moderation analysis, High status (US) vs. Lower status (US))

|  |  | **OR** | **99% CI** | |
| --- | --- | --- | --- | --- |
| **Fixed** | High status (US) (ref= Lower status, US) | 1.58 | .35 | 7.26 |
|  | Meritocracy score | 1.08 | .99 | 1.18 |
|  | High status (US)*Meritocracy score | .97 | .85 | 1.10 |
|  | Ladder score | 1.01 | .92 | 1.11 |
|  | High status (US)*Ladder score | 1.00 | .88 | 1.15 |
|  | Structural position | .98 | .58 | 1.65 |
|  | High status (US)*Structural position | .83 | .39 | 1.77 |
|  | Research Accomplishments | .79 | .47 | 1.32 |
|  | High status (US)*Research Accomplishments | .81 | .40 | 1.65 |
| **Random** | Discipline | .28 | .59 | 1.35 |
| **Model specification** | Number of disciplines | 6 |  |  |
|  | Number of respondents | 2543 |  |  |
|  | Log Likelihood | -1202.2983 |  |  |

**Table S39.** Mixed logit model predicting ‘Include in conference’ (Moderation analysis, High status (US) vs. Lower status (US))

|  |  | **OR** | **99% CI** | |
| --- | --- | --- | --- | --- |
| **Fixed** | High status (US) (ref= Lower status, US) | 0.42 | .11 | 1.60 |
|  | Meritocracy score | 1.06 | .98 | 1.15 |
|  | High status (US)*Meritocracy score | 1.03 | .92 | 1.16 |
|  | Ladder score | 0.97 | .92 | 1.16 |
|  | High status (US)*Ladder score | 1.05 | .94 | 1.19 |
|  | Structural position | .97 | .61 | 1.53 |
|  | High status (US)*Structural position | .90 | .46 | 1.76 |
|  | Research Accomplishments | .79 | .48 | 1.21 |
|  | High status (US)*Research Accomplishments | 1.06 | .55 | 2.04 |
| **Random** | Discipline | .84 | .18 | 3.78 |
| **Model specification** | Number of disciplines | 6 |  |  |
|  | Number of respondents | 2530 |  |  |
|  | Log Likelihood | -1498.0723 |  |  |

**Table S40.** Mixed tobit model predicting ‘abstract rating’

|  |  | **Coef.** | **SE** | **99% CI** | |
| --- | --- | --- | --- | --- | --- |
| **Fixed** | Intercept | 14.00 | 0.37 | 13.04 | 14.95 |
|  | High status, US | 0.03 | 0.10 | -0.22 | 0.28 |
|  | Lower status, Non-US | -0.01 | 0.10 | -0.27 | 0.24 |
|  | Lower status, US (Reference) |  |  |  |  |
|  |  |  |  |  |  |
| **Random** | Residual | 6.50 | 0.15 | 6.14 | 6.88 |
|  | Discipline | 0.80 | 0.47 | 0.18 | 3.61 |
|  |  |  |  |  |  |
| **Model specifications** | Number of disciplines | 6 |  |  |  |
|  | Number of respondents | 4111 |  |  |  |
|  | Log Likelihood | -9641.683 |  |  |  |

**Table S41.** Mixed tobit model predicting ‘Abstract rating’ (Manipulation check)

|  |  | **Coef.** | **SE** | **99% CI** | |
| --- | --- | --- | --- | --- | --- |
| **Fixed** | Intercept | 14.04 | 0.40 | 13.02 | 15.07 |
|  | High status, US | -0.06 | 0.13 | -0.38 | 0.27 |
|  | Lower status, Non-US | -0.08 | 0.12 | -0.38 | 0.23 |
|  | Lower status, US (Reference) |  |  |  |  |
|  |  |  |  |  |  |
| **Random** | Residual | 6.12 | 0.18 | 5.68 | 6.59 |
|  | Discipline | 0.91 | 0.53 | 0.20 | 4.12 |
|  |  |  |  |  |  |
| **Model specifications** | Number of disciplines | 6.00 |  |  |  |
|  | Number of respondents | 2474 |  |  |  |
|  | Log Likelihood | -5733.6237 |  |  |  |

**Table S42.** Mixed tobit model predicting ‘Abstract rating’ (Topic match)

|  |  | **Coef.** | **SE** | **99% CI** | |
| --- | --- | --- | --- | --- | --- |
| **Fixed** | Intercept | 14.05 | 0.42 | 12.96 | 15.14 |
|  | High status, US | 0.14 | 0.14 | -0.23 | 0.51 |
|  | Lower status, Non-US | 0.11 | 0.15 | -0.27 | 0.49 |
|  | Lower status, US (Reference) |  |  |  |  |
|  |  |  |  |  |  |
| **Random** | Residual | 6.95 | 0.22 | 6.40 | 7.55 |
|  | Discipline | 1.01 | 0.60 | 0.22 | 4.62 |
|  |  |  |  |  |  |
| **Model specifications** | Number of disciplines | 6 |  |  |  |
|  | Number of respondents | 1979 |  |  |  |
|  | Log Likelihood | -4707.24 |  |  |  |

**Table S43.** Mixed tobit model predicting ‘Abstract rating’ (Moderation analysis, Lower status (US) vs. Lower status (elsewhere))

|  |  | **Coef.** | **99% CI** | |
| --- | --- | --- | --- | --- |
| **Fixed** | Intercept | 11.99 | 10.52 | 13.47 |
|  | Lower status (US) (ref= Lower status, Non-US) | .23 | -1.24 | 1.71 |
|  | Meritocracy score | .19 | .10 | .28 |
|  | Lower status (US) *Meritocracy score | .014 | -.11 | .14 |
|  | Ladder score | -.01 | -.11 | .09 |
|  | Lower status (US) *Ladder score | -.07 | -.21 | .06 |
|  | Structural position | -.05 | -.58 | .49 |
|  | Lower status (US) *Structural position | .60 | -.14 | 1.35 |
|  | Research Accomplishments | -.03 | -.57 | .51 |
|  | Lower status (US) *Research Accomplishments | -.22 | -.97 | .53 |
| **Random** | Discipline | .94 | .21 | 4.28 |
|  | Residual | 6.25 | 5.81 | 6.74 |
| **Model specification** | Number of disciplines | 6 |  |  |
|  | Number of respondents | 2490 |  |  |
|  | Log Likelihood | -5798.3276 |  |  |

**Table S44.** Mixed tobit model predicting ‘Abstract rating’ (Moderation analysis, High status (US) vs. Lower status (US))

|  |  | **Coef.** | **99% CI** | |
| --- | --- | --- | --- | --- |
| **Fixed** | Intercept | 12.20 | 10.79 | 13.61 |
|  | High status (US) (ref= Lower status, US) | -.59 | -2.12 | .93 |
|  | Meritocracy score | .21 | .12 | .29 |
|  | High status (US) *Meritocracy score | -.00 | -.13 | .12 |
|  | Ladder score | -.08 | -.17 | .01 |
|  | High status (US) *Ladder score | .13 | .00 | .27 |
|  | Structural position | .56 | .05 | 1.07 |
|  | High status (US) *Structural position | -.61 | -1.36 | .139 |
|  | Research Accomplishments | -.28 | -.81 | .24 |
|  | High status (US)*Research Accomplishments | -.16 | -.90 | .58 |
| **Random** | Discipline | .81 | .18 | 3.68 |
|  | Residual | 6.32 | 5.87 | 6.89 |
| **Model specification** | Number of disciplines | 6 |  |  |
|  | Number of respondents | 2529 |  |  |
|  | Log Likelihood | -5900.1576 |  |  |

**Table S45. Completion rates by discipline.**

| **Discipline** | **Sent to** | **Started** | **Completed** |
| --- | --- | --- | --- |
| **Astronomy** | 12017 | 1143 (10%) | 762 (6%) |
| **Cardiology** | 24053 | 1074 (4%) | 748 (3%) |
| **Materials Science** | 23907 | 1493 (6%) | 804 (3%) |
| **Political Science** | 11325 | 1500 (13%) | 1308 (12%) |
| **Psychology** | 11938 | 1132 (9%) | 959 (8%) |
| **Public Health** | 12077 | 1059 (9%) | 832 (7%) |

Note. “Sent to” specifies the initial recruitment sample per discipline. “Started” specifies the number of scientists opening the survey. “Completed” specifies the number of participants completing the survey. The percentages in brackets do not account for the 15247 bounced emails.

**Table S46.** Distribution of scientists stratified by gender and age across the six disciplines in the full WoS population (population, the recruitment sample (sample) and the respondent sample (respondents)

| **Unit** | **Population** | | | | | |
| --- | --- | --- | --- | --- | --- | --- |
| **Gender** | **Women** | | **Men** | | **Unknown** | |
| **Field** | **%** | **Age** | **%** | **Age** | **%** | **Age** |
| astro | 17.9% | 14.2 | 65.4% | 18.1 | 16.7% | 14.7 |
| cardio | 25.6% | 11.1 | 61.8% | 14.5 | 12.6% | 11.9 |
| matsci | 16.5% | 11.3 | 60.4% | 14.3 | 23.1% | 10.4 |
| polsci | 28.3% | 11.9 | 62.2% | 14.8 | 9.4% | 12.3 |
| psych | 46.8% | 11.5 | 42.4% | 15.4 | 10.8% | 11.0 |
| pubhealth | 49.7% | 11.2 | 37.5% | 13.7 | 12.9% | 10.8 |
| **Unit** | **Sample** | | | | | |
| **Gender** | **Women** | | **Men** | | **Unknown** | |
| **Field** | **%** | **Age** | **%** | **Age** | **%** | **Age** |
| astro | 18.3% | 15.2 | 65.5% | 19.3 | 16.1% | 15.5 |
| cardio | 20.8% | 14.3 | 66.7% | 18.3 | 12.5% | 16.0 |
| matsci | 13.6% | 14.5 | 64.5% | 17.0 | 21.9% | 12.9 |
| polsci | 28.1% | 12.1 | 62.4% | 14.9 | 9.5% | 12.4 |
| psych | 44.6% | 12.4 | 44.8% | 16.7 | 10.6% | 12.3 |
| pubhealth | 50.9% | 12.8 | 36.4% | 16.1 | 12.7% | 12.9 |
| **Unit** | **Respondents** | | | | | |
| **Gender** | **Women** | | **Men** | | **Unknown** | |
| **Field** | **%** | **Age** | **%** | **Age** | **%** | **Age** |
| astro | 16.4% | 14.5 | 69.5% | 21.7 | 14.0% | 18.8 |
| cardio | 25.0% | 15.4 | 66.3% | 19.3 | 8.7% | 18.5 |
| matsci | 15.5% | 16.3 | 68.2% | 20.0 | 16.3% | 13.6 |
| polsci | 29.5% | 12.0 | 62.4% | 14.1 | 8.0% | 13.2 |
| psych | 46.0% | 13.0 | 45.5% | 17.5 | 8.6% | 13.4 |
| pubhealth | 52.7% | 13.7 | 37.8% | 17.4 | 9.6% | 14.5 |

**Table S47.** Distribution of scientists stratified by the top 30 countries with most authors in the full discipline-specific WoS populations, the recruitment samples and the respondent samples.

| Country | n_astro | sample_astro | resp_astro | n_cardio | sample_cardio | resp_cardio | n_matsci | sample_matsci | resp_matsci | n_polsci | sample_polsci | resp_polsci | n_psych | sample_psych | resp_psych | n_pubhealth | sample_pubhealth | resp_pubhealth |
| --- | --- | --- | --- | --- | --- | --- | --- | --- | --- | --- | --- | --- | --- | --- | --- | --- | --- | --- |
| usa | 10127 (29.7%) | 3005 (29.5%) | 124 (23.2%) | 18228 (26.1%) | 5652 (26.9%) | 119 (21.6%) | 8986 (24%) | 4159 (21.5%) | 66 (14.2%) | 5662 (37.9%) | 3661 (36.9%) | 301 (32.3%) | 18919 (38.1%) | 3886 (36.7%) | 150 (26.2%) | 21846 (36.8%) | 3765 (38.5%) | 194 (31.4%) |
| germany | 2505 (7.3%) | 712 (7%) | 49 (9.2%) | 4321 (6.2%) | 1434 (6.8%) | 35 (6.3%) | 3321 (8.9%) | 1893 (9.8%) | 63 (13.5%) | 825 (5.5%) | 565 (5.7%) | 50 (5.4%) | 3317 (6.7%) | 785 (7.4%) | 37 (6.5%) | 1606 (2.7%) | 290 (3%) | 14 (2.3%) |
| japan | 1813 (5.3%) | 508 (5%) | 12 (2.2%) | 6755 (9.7%) | 1460 (6.9%) | 7 (1.3%) | 3551 (9.5%) | 1649 (8.5%) | 15 (3.2%) | 69 (0.5%) | 33 (0.3%) | 3 (0.3%) | 851 (1.7%) | 136 (1.3%) | 4 (0.7%) | 1127 (1.9%) | 149 (1.5%) | 0 (0%) |
| england | 1873 (5.5%) | 598 (5.9%) | 28 (5.2%) | 2792 (4%) | 973 (4.6%) | 19 (3.4%) | 1421 (3.8%) | 815 (4.2%) | 20 (4.3%) | 1744 (11.7%) | 1216 (12.2%) | 48 (5.2%) | 3171 (6.4%) | 722 (6.8%) | 23 (4%) | 2625 (4.4%) | 511 (5.2%) | 29 (4.7%) |
| peoples r china | 1840 (5.4%) | 519 (5.1%) | 25 (4.7%) | 2757 (3.9%) | 706 (3.4%) | 5 (0.9%) | 4941 (13.2%) | 2267 (11.7%) | 33 (7.1%) | 150 (1%) | 99 (1%) | 4 (0.4%) | 1354 (2.7%) | 237 (2.2%) | 12 (2.1%) | 1455 (2.5%) | 194 (2%) | 5 (0.8%) |
| italy | 1780 (5.2%) | 561 (5.5%) | 39 (7.3%) | 5326 (7.6%) | 1350 (6.4%) | 56 (10.1%) | 730 (2%) | 449 (2.3%) | 23 (4.9%) | 294 (2%) | 198 (2%) | 30 (3.2%) | 1629 (3.3%) | 329 (3.1%) | 29 (5.1%) | 1540 (2.6%) | 211 (2.2%) | 13 (2.1%) |
| canada | 712 (2.1%) | 209 (2.1%) | 14 (2.6%) | 2459 (3.5%) | 742 (3.5%) | 11 (2%) | 578 (1.5%) | 282 (1.5%) | 12 (2.6%) | 680 (4.5%) | 418 (4.2%) | 40 (4.3%) | 2886 (5.8%) | 622 (5.9%) | 35 (6.1%) | 2757 (4.6%) | 502 (5.1%) | 34 (5.5%) |
| spain | 1136 (3.3%) | 324 (3.2%) | 21 (3.9%) | 2941 (4.2%) | 721 (3.4%) | 18 (3.3%) | 776 (2.1%) | 468 (2.4%) | 29 (6.2%) | 393 (2.6%) | 283 (2.9%) | 33 (3.5%) | 2618 (5.3%) | 586 (5.5%) | 35 (6.1%) | 1385 (2.3%) | 190 (1.9%) | 10 (1.6%) |
| france | 1903 (5.6%) | 589 (5.8%) | 38 (7.1%) | 2278 (3.3%) | 666 (3.2%) | 8 (1.4%) | 1830 (4.9%) | 1059 (5.5%) | 33 (7.1%) | 252 (1.7%) | 166 (1.7%) | 6 (0.6%) | 1567 (3.2%) | 362 (3.4%) | 17 (3%) | 1187 (2%) | 189 (1.9%) | 11 (1.8%) |
| australia | 767 (2.2%) | 224 (2.2%) | 9 (1.7%) | 1262 (1.8%) | 445 (2.1%) | 8 (1.4%) | 379 (1%) | 218 (1.1%) | 6 (1.3%) | 559 (3.7%) | 371 (3.7%) | 27 (2.9%) | 1685 (3.4%) | 373 (3.5%) | 19 (3.3%) | 2359 (4%) | 450 (4.6%) | 37 (6%) |
| netherlands | 659 (1.9%) | 220 (2.2%) | 19 (3.6%) | 2162 (3.1%) | 868 (4.1%) | 37 (6.7%) | 434 (1.2%) | 237 (1.2%) | 7 (1.5%) | 498 (3.3%) | 361 (3.6%) | 43 (4.6%) | 1580 (3.2%) | 317 (3%) | 15 (2.6%) | 1070 (1.8%) | 232 (2.4%) | 19 (3.1%) |
| brazil | 557 (1.6%) | 163 (1.6%) | 8 (1.5%) | 1612 (2.3%) | 472 (2.2%) | 11 (2%) | 287 (0.8%) | 179 (0.9%) | 7 (1.5%) | 82 (0.5%) | 49 (0.5%) | 6 (0.6%) | 531 (1.1%) | 113 (1.1%) | 9 (1.6%) | 3253 (5.5%) | 666 (6.8%) | 23 (3.7%) |
| poland | 396 (1.2%) | 125 (1.2%) | 9 (1.7%) | 1636 (2.3%) | 627 (3%) | 17 (3.1%) | 577 (1.5%) | 421 (2.2%) | 17 (3.6%) | 48 (0.3%) | 32 (0.3%) | 8 (0.9%) | 362 (0.7%) | 83 (0.8%) | 14 (2.4%) | 625 (1.1%) | 109 (1.1%) | 5 (0.8%) |
| sweden | 316 (0.9%) | 103 (1%) | 10 (1.9%) | 836 (1.2%) | 380 (1.8%) | 33 (6%) | 470 (1.3%) | 222 (1.1%) | 10 (2.1%) | 277 (1.9%) | 209 (2.1%) | 47 (5%) | 551 (1.1%) | 133 (1.3%) | 9 (1.6%) | 1108 (1.9%) | 252 (2.6%) | 38 (6.2%) |
| switzerland | 374 (1.1%) | 120 (1.2%) | 6 (1.1%) | 908 (1.3%) | 259 (1.2%) | 8 (1.4%) | 582 (1.6%) | 304 (1.6%) | 8 (1.7%) | 238 (1.6%) | 179 (1.8%) | 31 (3.3%) | 697 (1.4%) | 168 (1.6%) | 16 (2.8%) | 514 (0.9%) | 91 (0.9%) | 10 (1.6%) |
| india | 946 (2.8%) | 239 (2.3%) | 7 (1.3%) | 451 (0.6%) | 77 (0.4%) | 3 (0.5%) | 871 (2.3%) | 365 (1.9%) | 8 (1.7%) | 17 (0.1%) | 7 (0.1%) | 0 (0%) | 96 (0.2%) | 12 (0.1%) | 2 (0.3%) | 719 (1.2%) | 47 (0.5%) | 1 (0.2%) |
| russia | 922 (2.7%) | 281 (2.8%) | 11 (2.1%) | 594 (0.9%) | 120 (0.6%) | 4 (0.7%) | 1244 (3.3%) | 796 (4.1%) | 15 (3.2%) | 52 (0.3%) | 25 (0.3%) | 3 (0.3%) | 182 (0.4%) | 36 (0.3%) | 5 (0.9%) | 82 (0.1%) | 7 (0.1%) | 1 (0.2%) |
| turkey | 159 (0.5%) | 53 (0.5%) | 4 (0.7%) | 1993 (2.9%) | 751 (3.6%) | 13 (2.4%) | 237 (0.6%) | 160 (0.8%) | 6 (1.3%) | 112 (0.7%) | 75 (0.8%) | 10 (1.1%) | 238 (0.5%) | 53 (0.5%) | 4 (0.7%) | 206 (0.3%) | 27 (0.3%) | 1 (0.2%) |
| south korea | 351 (1%) | 109 (1.1%) | 1 (0.2%) | 808 (1.2%) | 286 (1.4%) | 1 (0.2%) | 1075 (2.9%) | 475 (2.5%) | 3 (0.6%) | 130 (0.9%) | 79 (0.8%) | 4 (0.4%) | 250 (0.5%) | 52 (0.5%) | 1 (0.2%) | 294 (0.5%) | 45 (0.5%) | 0 (0%) |
| belgium | 314 (0.9%) | 96 (0.9%) | 12 (2.2%) | 758 (1.1%) | 263 (1.3%) | 11 (2%) | 219 (0.6%) | 144 (0.7%) | 1 (0.2%) | 206 (1.4%) | 149 (1.5%) | 18 (1.9%) | 697 (1.4%) | 178 (1.7%) | 9 (1.6%) | 386 (0.7%) | 74 (0.8%) | 6 (1%) |
| denmark | 165 (0.5%) | 49 (0.5%) | 4 (0.7%) | 775 (1.1%) | 336 (1.6%) | 46 (8.3%) | 169 (0.5%) | 81 (0.4%) | 5 (1.1%) | 226 (1.5%) | 176 (1.8%) | 38 (4.1%) | 240 (0.5%) | 57 (0.5%) | 8 (1.4%) | 667 (1.1%) | 136 (1.4%) | 24 (3.9%) |
| israel | 177 (0.5%) | 58 (0.6%) | 4 (0.7%) | 555 (0.8%) | 186 (0.9%) | 5 (0.9%) | 330 (0.9%) | 141 (0.7%) | 1 (0.2%) | 160 (1.1%) | 108 (1.1%) | 9 (1%) | 652 (1.3%) | 148 (1.4%) | 12 (2.1%) | 230 (0.4%) | 36 (0.4%) | 5 (0.8%) |
| taiwan | 210 (0.6%) | 63 (0.6%) | 0 (0%) | 452 (0.6%) | 175 (0.8%) | 1 (0.2%) | 678 (1.8%) | 361 (1.9%) | 1 (0.2%) | 45 (0.3%) | 25 (0.3%) | 3 (0.3%) | 278 (0.6%) | 51 (0.5%) | 2 (0.3%) | 252 (0.4%) | 40 (0.4%) | 0 (0%) |
| portugal | 117 (0.3%) | 40 (0.4%) | 3 (0.6%) | 812 (1.2%) | 177 (0.8%) | 4 (0.7%) | 75 (0.2%) | 49 (0.3%) | 3 (0.6%) | 73 (0.5%) | 54 (0.5%) | 12 (1.3%) | 530 (1.1%) | 114 (1.1%) | 16 (2.8%) | 251 (0.4%) | 53 (0.5%) | 11 (1.8%) |
| norway | 82 (0.2%) | 26 (0.3%) | 4 (0.7%) | 467 (0.7%) | 208 (1%) | 15 (2.7%) | 82 (0.2%) | 52 (0.3%) | 2 (0.4%) | 221 (1.5%) | 161 (1.6%) | 27 (2.9%) | 383 (0.8%) | 96 (0.9%) | 6 (1%) | 584 (1%) | 131 (1.3%) | 25 (4.1%) |
| scotland | 269 (0.8%) | 81 (0.8%) | 4 (0.7%) | 309 (0.4%) | 113 (0.5%) | 2 (0.4%) | 172 (0.5%) | 107 (0.6%) | 1 (0.2%) | 238 (1.6%) | 173 (1.7%) | 8 (0.9%) | 388 (0.8%) | 94 (0.9%) | 6 (1%) | 424 (0.7%) | 83 (0.8%) | 4 (0.6%) |
| finland | 220 (0.6%) | 70 (0.7%) | 7 (1.3%) | 361 (0.5%) | 155 (0.7%) | 11 (2%) | 182 (0.5%) | 124 (0.6%) | 8 (1.7%) | 105 (0.7%) | 74 (0.7%) | 16 (1.7%) | 298 (0.6%) | 73 (0.7%) | 6 (1%) | 511 (0.9%) | 109 (1.1%) | 16 (2.6%) |
| iran | 153 (0.4%) | 50 (0.5%) | 0 (0%) | 259 (0.4%) | 90 (0.4%) | 0 (0%) | 228 (0.6%) | 123 (0.6%) | 4 (0.9%) | 6 (0%) | 4 (0%) | 0 (0%) | 113 (0.2%) | 19 (0.2%) | 1 (0.2%) | 713 (1.2%) | 94 (1%) | 3 (0.5%) |
| austria | 202 (0.6%) | 67 (0.7%) | 6 (1.1%) | 437 (0.6%) | 139 (0.7%) | 5 (0.9%) | 265 (0.7%) | 157 (0.8%) | 4 (0.9%) | 125 (0.8%) | 80 (0.8%) | 14 (1.5%) | 294 (0.6%) | 72 (0.7%) | 4 (0.7%) | 132 (0.2%) | 22 (0.2%) | 3 (0.5%) |
| greece | 132 (0.4%) | 48 (0.5%) | 6 (1.1%) | 830 (1.2%) | 246 (1.2%) | 10 (1.8%) | 99 (0.3%) | 72 (0.4%) | 2 (0.4%) | 55 (0.4%) | 36 (0.4%) | 5 (0.5%) | 128 (0.3%) | 29 (0.3%) | 1 (0.2%) | 162 (0.3%) | 27 (0.3%) | 0 (0%) |

**Table S48** Variable specifications

|  | **Explanation** | **Measurement type** |
| --- | --- | --- |
| **Explanatory variables** |  |  |
| High status, US | Subjects exposed to an abstract from a high status US university=1, all others=0 | Dummy (0,1) |
| Low status, US | Subjects exposed to an abstract from a lower status US university=1, all others=0 | Dummy (0,1) |
| Low status, Non-US | Subjects exposed to an abstract from a lower status non-US university=1, all others=0 | Dummy (0,1) |
| **Dependent variables** |  |  |
| Abstract rating | Composite measure of four items asking about the originality, credibility, significance and clarity of the presented abstract. | Count (4-20) |
| Open full-text | Measures whether or not the participant would consider opening the full text after reading the abstract (opening full-text=1). | Dummy (0,1) |
| Include in conference | Measures whether or not the participant would consider including the abstract for an oral presentation in a conference program (including abstract=1). | Dummy (0,1) |
| **Moderators** |  |  |
| Meritocratic beliefs | Composite measure of ‘meritocratic beliefs’ of three items about the participants’ descriptive beliefs in the objectivity and fairness of peer-evaluation in their own research fields. | Count (3-15) |
| Structural location | Measure of the participants’ structural location in the science system in terms of scientific rank and institutional affiliation (Associate professors, full professors, chairs and deans affiliated with a top university = 1. All others = 0). | Dummy (0,1) |
| Research accomplishments | Measures the participants’ own research accomplishments in terms of citation impact (Among the top 10% most cited in Web of Science = 1. Not among the top 10% most cited in Web of Science = 0). | Dummy (0,1) |
| Self-perceived status in field | Measures the participants’ self-perceived status in their research area. | Count (0-9) |
| **Control variables** |  |  |
| Manipulation check | Subjects responding correctly to the manipulation check=1. Subjects responding incorrectly to the manipulation check=0. | Dummy (0,1) |
| Perceived research distance from abstract | Measure of how close the subject addressed in the abstract is to the participant’s own work (‘Not close at all’, ‘Not too close’=0, ‘Somewhat close’, ‘Very close’, ‘Extremely close’ = 1). | Dummy (0, 1) |

**Supplementary Table 49** University and country affiliations listed in the abstracts

| **Discipline** | **High-status (US)** | **Low-status university (US)** | **Low status (non-US)** |
| --- | --- | --- | --- |
| **Astronomy** | Harvard University  UC Berkeley  Columbia University  M.I.T.  California Inst. of Tech. | Brigham Young University Clemson University  University of Texas Arlington  University of Oklahoma  University of Missouri | Ben Gurion University of the Negev (Israel)National and Kapodistrian University of Athens (Greece)Universidade Federal de Santa Catarina (Brazil)Complutense University (Spain)Yonsei University (South Korea) |
| **Cardiology** | Harvard University  Stanford University  Columbia University  Johns Hopkins University  Yale University | Florida International University  University of Central Florida  University of Toledo  Southern Illinois University- Carbondale  East Carolina University | University of Tartu (Estonia)  University of the Witwatersrand (South Africa)  University of Chile (Chile)  Masaryk University (Czech Republic)  National and Kapodistrian University of Athens (Greece) |
| **Materials Science** | Stanford University  Harvard University  M.I.T.  UC Berkeley  California Inst. of Tech. | Brigham Young University Clemson University University of Texas Arlington  University of Oklahoma University of Missouri | Budapest University of Technology and Economics (Hungary)Universidade Federal de Santa Catarina (Brazil)University of Coimbra (Portugal)Indian Institute of Science (India)Ben-Gurion University of the Negev (Israel) |
| **Political Science** | Stanford University  Harvard University  M.I.T.  UC Berkeley  Yale University | East Carolina University  University of North Texas  Kansas State University  Louisiana State University  Southern Illinois University | Yonsei University (South Korea)  Masaryk University (Czech Republic)  Jagiellonian University (Poland)  University of Chile (Chile)  Sogang University (South korea) |
| **Psychology** | Stanford University  Harvard University  Princeton University  Yale University  Columbia University | East Carolina University,  University of North Texas  Kansas State University  Louisiana State University  Southern Illinois University–Carbondale | Yonsei University (South Korea)  Masaryk University (Czech Republic)  Jagiellonian University (Poland)  University of Chile (Chile)  Sogang University (South korea) |
| **Public Health** | Harvard University  Johns Hopkins University  UC Berkeley  Columbia University  Yale University | Florida International University  University of Central Florida  University of Toledo  Southern Illinois University- Carbondale  East Carolina University | University of Tartu, (Estonia)  University of the Witwatersrand (South Africa)  University of Tartu (Estonia)  Masaryk University (Czech Republic)  National and Kapodistrian University of Athens (Greece) |
